# Reasons for betel quid chewing amongst dependent and non-dependent betel quid chewing adolescents: a school-based cross-sectional survey

**DOI:** 10.1186/s13011-018-0154-5

**Published:** 2018-05-09

**Authors:** Azmina Hussain, Sidra Zaheer, Kashif Shafique

**Affiliations:** 1 Dr. Ishrat-ul-Ibad Khan Institute of Oral Health Sciences, Dow University of Health Sciences, OJHA Campus, SUPARCO road, Gulzar e Hijri, Scheme 33, Karachi, Pakistan; 2 School of Public Health, Dow University of Health Sciences, OJHA Campus, SUPARCO road, Gulzar e Hijri, Karachi, Pakistan; 3 Institute of Health and Wellbeing, Public Health, University of Glasgow, 1-Lilybank Gardens, Glasgow, G12 8RZ UK

**Keywords:** Dependency, Betel quid dependence scale, Reasons for betel quid chewing, Adolescents

## Abstract

**Background:**

Betel quid (BQ) chewing in children is initiated in their adolescence. It is pivotal to understand adolescents’ reasons behind chewing BQ. In this study, we aimed to evaluate the reasons for BQ chewing amongst adolescents using reasons for betel quid chewing scale (RBCS) and their associated dependency on it.

**Methods:**

This is a cross-sectional school based survey. Out of 2200 adolescents from 26 schools of Karachi, 874 BQ chewers were assessed for their reasons of BQ chewing and dependency on it. Regression analyses were employed to report crude and adjusted (after adjusting for all reasons of BQ chewing) effect sizes with 95% confidence interval and *P*-value was set significant at < 0.05.

**Results:**

Students who believed that BQ chewing relaxes them (stimulation construct) were twice as likely to be dependent on BQ (OR = 2.36, 95% CI (1.20–4.65) as compared with others. Participants who thought it eases their decision making (stimulation construct), were sizably more likely to be dependent on BQ (OR = 9.65, 95% CI (4.15–22.43) than those who did not consider ease in decision making important. Adolescents who considered not chewing as rude (social/cultural construct), were thrice more likely to be dependent on BQ (OR = 2.50, 95% CI (1.11–5.63) than others.

**Conclusions:**

Stimulation remained fundamental chewing reason followed by social/cultural trigger amongst adolescents. Any future intervention may get favorable results if it addresses ways to overcome stimulation and social/cultural barriers that are strongly associated with BQ chewing and dependency.

## Background

Globally, approximately 600 million people chew betel quid (BQ). Three fourth of its consumption is concentrated in the Pacific Islands, South Asian, and Southeast Asian countries [1]. This habit commences at a very early age of nearly 13 years thus rendering adolescents as a high risk group [2]. After alcohol, caffeine, and nicotine; betel quid is yet another substance used worldwide [3–5]. Betel quid has a known relaxing and stimulant effect by acting on autonomic nervous system [6]. The formulation of betel quid depends on a variety of factors which may include cultural background of a country, regional backdrop, personal likings and availability of specific ingredients. It is typically made up of betel leaf, betel/areca nut, slaked lime with or without tobacco [7]. The frequently available products in Pakistan are; paan masala, paan (betel nut with betel leaf with or without tobacco), chaaliya, supari, gutka (which also has synthetic flavored scent), other forms of smokeless tobacco which have betel nut in it, cardamom, catechu and/or lime [4].

Though the use of betel quid has a proven association with oral cancers and potentially malignant lesions like oral sub mucus fibrosis, and leukoplakia [8], and betel quid has been classified as a Group 1 carcinogen by the International Agency for Research on Cancer [7, 9]; yet most researches so far have been focusing on its prevalence and genetic aspects [10, 11]. And a very limited work has been done on it psychological and behavioral component. It is vital to understand the reasons behind chewing these products so to develop interventions that may help to cease the habit and minimize related disease burden [12]. Social sciences studies suggest that human behaviors are incentive driven [13], such as they may chew betel nut as they like the way it makes them feel or they like the taste. After chronic use, they become dependent on it due to arecoline [14], nicotine [15] and also tobacco components. There may be several functional, behavioral, ethnic and communal motives behind substance abuse and limited work has been done in its pursuance. A study on cab drivers was conducted which suggested similar reasons of chewing as of smoking [16]. Followed by which a standardized instrument to record reasons for BQ chewing was tested by Little et al. [12] in 2014. Both the studies focused on adult population. To date, ascertainment of the reasons for BQ chewing by a standard instrument in adolescents has not been assessed. We aimed to determine reasons of BQ chewing in adolescents by using Reasons for Betel Quid Chewing Scale (RBCS) along with identifying the reasons that affect the dependency on BQ which will be measured by betel quid dependency scale (BQDS).

## Methods

### Data source

With 24 million population, Karachi is rendered as the largest city of Pakistan [17] that has 06 districts and 18 towns [18]. Adolescents from 26 government and private schools of Karachi were assessed for their reasons of chewing BQ and for their dependency on it.

### Sampling and study participants

The government and private schools were selected based on cluster sampling by proportionate recruitment of both types of schools. In openEpi, a sample size of 1606 was calculated and increased to 2200 after adding attrition rate. The number and size of each cluster was deliberated by using manual equations as software was unavailable [19]. A substantially representative sample of 2200 adolescents (aged 11–16 years) from grade VI-X were enrolled with 50–100 students per school after attaining consent from schools’ principals and parents.

### Data collection tool and study variables

#### Reasons for betel-quid chewing scale – RBCS [12]

Based on the reasons for smoke [20–22], RBCS was developed and validated first in Taiwanese cab drivers and then in a small sample of Guam male and female users. In this study, we used RBCS on adolescents. This is a 10-items scale to measure reasons of betel quid chewing. Three-point scale was used to record responses (0-not important, 1-neutral, and 2-important). Three factors overarched 10 items namely; reinforcement construct (this measured the likeness of consumers for the taste of the product and if they like to keep something in their mouth always), social/cultural construct (this included BQ use by family and friends along with cultural connotation attached to the use), and stimulation factor (this evaluated the level of psychological effects on an individual as a rationale behind chewing).

#### Betel quid dependence scale – BQDS [23]

Lee et al. in 2012 developed a new instrument to assess betel quid dependency as BQDS [24]. The BQDS was initially conducted on male prisoners (who were ex chewers) in Chinese language [24]. Second study was conducted in English language and included both adult genders [23].

This is a 16-items based scale divided in 3 factors to determine dependency on BQ. There are 16 items in the scale under 3 factors; “physical and psychological urgent need,” “increasing dose,” and “maladaptive use”. Each item has two responses (Yes = 1 and No =0). Coded scores are between 0 and 1 and 0.5 score is deliberated as endorsement of half of the scale thus labelling an individual as ‘dependent’ on BQ [23]. A cut off of 4 was considered as “dependency on BQ” in adults in a study [25]. Since adolescents tend to overstate their dependency on BQ [26], we used a cautious approach by keeping 0.5 BQDS criterion score (8 out of 16 items) to define dependency in adolescents [27].

### Betel quid intake

Three questions were asked to establish betel quid consumption by these individuals; how many packets do they chew each day, since how many years they have been chewing and which type of BQ they use (only areca nut that is paan masala, BQ without tobacco and BQ with tobacco).

All questionnaires were translated into local language (Urdu) and back to English to ensure harmony.

### Ethical considerations

The ethical approval of this study was granted by Institutional Review Board of Dow University of Health Sciences, (Reference Number: IRB-725/DUHS/Approval/2016/219).

### Statistical analysis

Data of the current study were analyzed by using SPSS v22. Means, standard deviations and factor loadings of each item of RBCS were reported in descriptive forms. Also, chi-square was employed for the analysis of associations between reasons for BQ use and dependency variable. Univariate and multivariate binary logistic regression analyses were used to ascertain the associations between all study variables (BQ consumption and RBCS items) and dependency on BQ. We assessed the differences in reasons for chewing between the dependent chewing group and non-dependent chewing group. Results were reported in both crude and adjusted odds ratio after adjustments of all variables at 95% confidence interval and *P*-value was set significant at < 0.05.

## Results

### Basic characteristics of study sample

Out of 2200 children, 2140 provided complete information amongst whom 874 were found to be using BQ in any form (i.e. areca nut only in the form of paan masala, betel quid without tobacco or betel quid with tobacco additives) at least once in past 30 days. Amongst 874 users, areca nut and paan masala users were 95.7% (*n* = 837), while 2.86% (*n* = 25) and 1.37% (*n* = 12) were BQ users without and with tobacco respectively.

### Mean, standard deviations and endorsement of items of RBCS

Table 1 displays 10 items of RBCS loaded in three factors. Highest mean scores and percentages per item suggested most frequently endorsed reason for chewing. For 68.9% of the adolescents, the taste of BQ formulations was most important reason for chewing [I like the taste – mean score 1.51(SD = 0.77)] while second most important reason was that 31.9% of them liked to have something in their mouth always [mean score 0.72(SD = 0.91)]. These were items of reinforcement factor whereas social/cultural and stimulation factors were least endorsed reasons of chewing for this group.

**Table 1:** Frequency of items endorsed on Reasons of Betel Quid Use amongst Adolescents (*n* = 874)

Items of RBCS	N (%)	Mean (SD)
Reinforcement construct		
I like the taste		1.51 (0.77)
Its not important	154 (17.6%)	
Neutral	118 (13.5%)	
Important	602 (68.9%)	
I like to have something in my mouth at all time		0.72 (0.91)
Its not important	522 (59.7%)	
Neutral	073 (8.4%)	
Important	279 (31.9%)	
Social/cultural construct		
All of my friends chew		0.65 (0.90)
Its not important	561 (64.2%)	
Neutral	059 (6.8%)	
Important	254 (29.1%)	
My family members chew		0.21 (0.59)
Its not important	720 (89.4%)	
Neutral	016 (2.0%)	
Important	069 (8.6%)	
Its rude not to chew		0.24 (0.63)
Its not important	717 (89.1%)	
Neutral	010 (1.2%)	
Important	078 (9.7%)	
People will not respect me if I don’t chew		0.12 (0.44)
Its not important	750 (93.2%)	
Neutral	017 (2.1%)	
Important	038 (4.7%)	
Stimulation construct		
It relaxes me		0.40 (0.78)
Its not important	650 (80.7%)	
Neutral	030 (3.7%)	
Important	125 (15.5%)	
It gives me energy		0.14 (0.50)
Its not important	756 (93.9%)	
Neutral	009 (1.1%)	
Important	040 (5.0%)	
It helps me make decisions		0.21 (0.59)
Its not important	742 (92.2%)	
Neutral	011 (1.4%)	
Important	052 (6.5%)	
I like the way it makes me feel		0.46 (0.82)
Its not important	624 (77.5%)	
Neutral	025 (3.1%)	
Important	156 (19.4%)	

*RBCS* Reasons for betel quid chewing scale, *SD* Standard deviation

### Reasons for chewing BQ for dependent and non-dependent chewers

BQDS suggested that 7.9% (*n* = 69) of the total users were dependent on BQ (any form) while 92.1% (*n* = 805) formulated the non-dependent chewing group (Table 2).

**Table 2:** Reasons of Betel Quid Use amongst dependent and non-dependent chewers (*n* = 874)

Reasons for Betel Quid Chew	No Dependency	Dependency	*p*-value	Chi-Square
	*n* = 805 (92.1%)	n = 69 (7.9%)		
Reinforcement construct				
I like the taste				
Its not important	144 (17.9%)	010 (14.5%)	0.304	2.38
Neutral	112 (13.9%)	006 (8.7%)		
Important	549 (68.2%)	053 (76.8%)		
I like to have something in my mouth at all time				
Its not important	495 (61.5%)	027 (39.1%)	< 0.001	20.96
Neutral	070 (8.7%)	003 (4.3%)		
Important	240 (29.8%)	039 (56.5%)		
Social/cultural construct				
All of my friends chew				
Its not important	524 (65.1%)	037 (53.6%)	0.016	8.30
Neutral	057 (7.1%)	002 (2.9%)		
Important	224 (27.8%)	030 (43.5%)		
My family members chew				
Its not important	720 (89.4%)	55 (79.7%)	0.019	7.91
Neutral	016 (2.0%)	001 (1.4%)		
Important	069 (8.6%)	013 (18.8%)		
Its rude not to chew				
Its not important	717 (89.1%)	047 (68.1%)	< 0.001	25.56
Neutral	010 (1.2%)	002 (2.9%)		
Important	078 (9.7%)	020 (29.0%)		
People will not respect me if I don’t chew				
Its not important	750 (93.2%)	063 (91.3%)	0.836	0.35
Neutral	017 (2.1%)	002 (2.9%)		
Important	038 (4.7%)	004 (5.8%)		
Stimulation construct				
It relaxes me				
Its not important	650 (80.7%)	032 (46.4%)	< 0.001	52.74
Neutral	030 (3.7%)	002 (2.9%)		
Important	125 (15.5%)	035 (50.7%)		
It gives me energy				
Its not important	756 (93.9%)	051 (73.9%)	< 0.001	40.51
Neutral	009 (1.1%)	001 (1.4%)		
Important	040 (5.0%)	017 (24.6%)		
It helps me make decisions				
Its not important	742 (92.2%)	035 (50.7%)	< 0.001	1.14
Neutral	011 (1.4%)	003 (4.3%)		
Important	052 (6.5%)	031 (44.9%)		
I like the way it makes me feel				
Its not important	624 (77.5%)	034 (49.3%)	< 0.001	28.57
Neutral	025 (3.1%)	003 (4.3%)		
Important	156 (19.4%)	032 (46.4%)		

Significant *P*-value – 0.05

### Univariate regression analysis

#### Reinforcement construct

The individuals who always liked to have something in their mouth were found to be significantly associated **(**χ2 = 20.96, df = 2, *p*-value < 0.001) with BQ dependency. Univariate analysis suggested that individuals who liked to have something in their mouths always, were thrice more likely to be dependent on BQ (OR = 2.97, 95% CI: 1.78–4.98), as compared with those who did not want something in their mouths always.

#### Social construct

Regarding social construct; out of four items, three (if their friends chewed, if their parents/family members chewed and when they thought it is rude not to chew BQ) were associated with BQ dependency. Participants who believed it to be important if their friends and family members chewed were nearly 2 times (OR = 1.89, 95% CI: 1.14–3.14) and 2.5 times (OR = 2.46, 95% CI: 1.28–4.73) respectively more likely to be dependent on BQ than those for whom it was not important if their family or friends chewed BQ.

Adolescents who considered not chewing BQ as rudeness were 4 times more likely to be dependent on BQ (OR = 3.91, 95% CI: 2.20–6.93) as compared with those chewers who did not consider it to be an important reason of chewing.

#### Stimulation construct

BQ chewing relaxed individuals more and this was significantly associated with dependency on BQ **(**χ2 = 52.74, df = 2, *p*-value < 0.001) (Table 2). Students who firmly believed that BQ chewing relaxed them were approximately six times more likely to be dependent on BQ (OR = 5.68, 95% CI: 3.39–9.53) as compared with others.

Participants for whom ease in decision making was of importance, their dependency was considerably likely to be high (OR = 12.63, 95% CI: 7.22–22.10) as compared with those who did not consider it to be a motive behind their chewing.

Students also liked the way BQ made them feel and this reason made them 4 times more likely to be dependent on it (OR = 3.76, 95% CI: 2.25–6.29) as compared with those who did not consider it to be important.

### Multivariate regression analysis

#### Reinforcement construct

Significant univariate finding regarding I like to have something in mouth always, became non-significant after multivariate analysis (Table 3).

**Table 3:** Level of dependency on betel quid and its use reasons among BQ Users (*n* = 874)

Betel Quid Dependency				
	OR (95% CI)	*p*-value	aOR (95% CI)	*p*-value
Reinforcement construct				
I like the taste				
Its not important	1		1	
Neutral	0.77 (0.27–2.18)	0.625	0.60 (0.18–1.93)	0.395
Important	1.39 (0.69–2.80)	1.390	0.76 (0 .34–1.69)	0.509
I like to have something in my mouth at all time				
Its not important	1		1	
Neutral	0.78 (0.23–2.65)	0.698	0.36 (0.08–1.52)	0.169
Important	2.97 (1.78–4.98)	< 0.001	0.96 (0.48–1.95)	0.927
Social/cultural construct				
All of my friends chew				
Its not important	1		1	
Neutral	0.49 (0.11–2.11)	0.344	0.33 (0.06–1.58)	0.166
Important	1.89 (1.14–3.14)	0.013	0.85 (0.44–1.67)	0.655
My family members chew				
Its not important	1		1	
Neutral	0.81 (0.10–6.28)	0.847	0.39 (0.03–4.11)	0.435
Important	2.46 (1.28–4.73)	0.007	0.99 (0.41–2.35)	0.986
Its rude not to chew				
Its not important	1		1	
Neutral	3.05 (0.65–14.32)	0.157	3.91 (0.66–23.15)	0.132
Important	3.91 (2.20–6.93)	< 0.001	2.50 (1.11–5.63)	0.027
People will not respect me if I don’t chew				
Its not important	1		1	
Neutral	1.40 (0.31–6.19)	0.657	0.39 (0.06–2.48)	0.32
Important	1.25 (0.43–3.62)	0.677	0.36 (0.09–1.35)	0.132
Stimulation construct				
It relaxes me				
Its not important	1		1	
Neutral	1.35 (0.3105.91)	0.687	0.66 (0.12–3.49)	0.63
Important	5.68 (3.39–9.53)	< 0.001	2.36 (1.20–4.65)	0.012
It gives me energy				
Its not important	1		1	
Neutral	1.64 (0.20–13.25)	0.639	0.40 (0.02–5.89)	0.507
Important	6.30 (3.34–11.88)	< 0.001	1.89 (0.81–4.42)	0.138
It helps me make decisions				
Its not important	1		1	
Neutral	5.78 (1.54–21.66)	0.009	4.57 (0.86–24.12)	0.073
Important	12.63 (7.22–22.10)	< 0.001	9.65 (4.15–22.43)	< 0.001
I like the way it makes me feel				
Its not important	1		1	
Neutral	2.20 (0.63–7.65)	0.214	2.29 (0.52–10.01)	0.270
Important	3.76 (2.25–6.29)	< 0.001	0.78 (0.35–1.75)	0.560

*BQ* Betel Quid, *OR* odds ratios, *aOR* adjusted odds ratios, *CI* confidence interval

#### Social construct

It is important to chew BQ if family members or friends chew became non-significant finding after multivariate adjustments of remaining BQ chewing reasons (Table 3). It is rude not to chew BQ remained significant reason of BQ chewing even after the adjustments for the remaining reasons of RBCS (OR = 2.50, 95% CI: 1.11–5.63) (Table 3).

#### Stimulation construct

The effect of the reason that BQ relaxes individuals persisted even after adjustments for remaining reasons of RBCS (OR = 2.36, 95% CI: 1.20–4.65) (Table 3). BQ chewing eases decision making remained significantly enormous even after adjustments for the remaining reasons (OR = 9.65, 95% CI: 4.15–22.43). I like the way it makes me feel became non-significant finding followed by multivariate analysis (Table 3).

## Discussion

In this study, the stimulation construct was found to be the most significantly marked rationale for chewing BQ. The significant findings of stimulation factor amongst BQ users (who were BQ dependent) included that BQ relaxed them and helped them in making decisions. This was followed by social/cultural factor where students considered it to be rude not to chew if their friends or family members were chewing BQ. The least frequently endorsed RBCS factor was reinforcement factor which suggested that children liked the taste of the BQ and they wanted something in their mouths always to chew.

The ‘stimulation’ (BQ in any form relaxed individuals in this study and eased their decision making) was the major reason of BQ chewing amongst dependent individuals and was coherent with study on reasons of smoking amongst adolescents may be because of nicotine [28] or combined effect of both nicotine and arecoline [15]. In our study, BQ relaxed adolescents and this made them six times more dependent on it than those who were non-dependent. This was a profound finding in other studies on motives behind smoking in both adults and adolescents [29, 30]. Focus on stimulation factor in future interventions for BQ use cessation and reduction in addiction may play a phenomenal role. For instance, the cognitive-behavioral therapy (CBT) approach to help smokers in quitting [31], may be modified and customized for BQ use cessation.

The second most commonly endorsed factor amongst dependent group was ‘social/cultural construct’ of RBCS. In current study, this suggested that a substantial chewing in adolescents was prompted by the company of a friend or a family member who chewed BQ and, when children thought it was rude if they did not chew. This was a signpost that amongst adolescents it was very important to initiate and then subsequently increase the use of BQ to be accepted as a member of the friend circle thus becoming a part of greater pursuit of societal connections. This finding was comprehensible with other studies on smoker adolescents [28] and BQ chewing adults [12, 32] as well as teenagers [33]. As a resultant effect, future interventions can be designed which may include strategies to cope with these societal norms, together with how to stay away from these chewing habits and yet be a part of the group. Also, smoking cessation interventions which were social-influence based and which proved to be effective in adolescents, can also be adapted to alter BQ chewing patterns [12].

The third and last important construct of RBCS was ‘reinforcement’ which was uniformly endorsed by both dependent and non-dependent groups in the scale (RBCS) thus suggesting that the initiation of habit is triggered by the taste of the BQ formulations. Like teenagers from other study marked pleasure from smoking as their motivational factor to initiate smoking [28], similarly adolescents of this study promoted the taste of BQ as a chewing trigger.

As per our knowledge, this is the first ever study to focus on the reasons of betel quid chewing in adolescents in a large representative sample of Pakistani schools; alongside, comparing the difference in reasons of BQ chewing between dependent and non-dependent groups. RBCS is first time ever used in this study for adolescents while earlier it was only used in adults [12, 16].

The limitations of this study include self-reported data whose quality may have been compromised by over or understatement of the participants regarding their perceived reasons of chewing BQ. Another limitation of such surveys may include recall bias but it might not have influenced our study, as these were reasons of why they chew BQ and there was nothing substantial to recall about past exposures. The effect of tobacco in betel quid can confound the effect of areca nut but since in our study only 1.3% individuals used BQ with tobacco this is unlikely to have any considerable effect on the overall findings of this study.

## Conclusion

Stimulation was the major construct of the RBCS that was fundamental chewing reason amongst the dependent chewing group followed by which was social/cultural trigger that initiated and augmented the chewing habit amongst adolescents. Any future intervention which focuses on stimulation and social/cultural reasons of BQ chewing, may get favorable results in ceasing the BQ chewing habit amongst adolescents thereby reducing related disease burden.

## Abbreviations

aOR: Adjusted odds ratio
BQ: Betel Quid
BQDS: Betel quid dependency scale
CI: Confidence interval
OR: Odds ratio
RBCS: Reasons for betel quid chewing scale
SD: Standard deviation

## References

[1] Auluck A, Hislop G, Poh C, Zhang L, Rosin M (2009). Areca nut and betel quid chewing among south Asian immigrants to western countries and its implications for oral cancer screening. Rural Remote Health.

[2] Sorensen G, Gupta PC, Sinha DN, Shastri S, Kamat M, Pednekar MS (2005). Teacher tobacco use and tobacco use prevention in two regions in India: qualitative research findings. Prev Med.

[3] Boucher BJ, Mannan N (2002). Metabolic effects of the consumption of Areca catechu. Addict Biol.

[4] Warnakulasuriya S, Peters T (2002). Introduction: biology, medical and socio-economic aspects of areca nut use. Addict Biol.

[5] Mazahir S, Malik R, Maqsood M, Merchant KA, Malik F, Majeed A (2006). Socio-demographic correlates of betel, areca and smokeless tobacco use as a high risk behavior for head and neck cancers in a squatter settlement of Karachi, Pakistan. Subst Abuse Treat Prev Policy.

[6] Winstock A (2002). Areca nut-abuse liability, dependence and public health. Addict Biol.

[7] IARC Working Group on the Evaluation of Carcinogenic Risks to Humans, World Health Organization, International Agency for Research on Cancer. Betel-quid and areca-nut chewing and some areca-nut-derived nitrosamines. IARC; 2004. PMC4781453 15635762

[8] Zaw K-K, Ohnmar M, Hlaing M-M, Win S-S, Aye P-P, Shwe S (2016). Betel quid and oral potentially malignant disorders in a periurban township in Myanmar. PLoS One.

[9] Lin C-F, Wang J-D, Chen P-H, Chang S-J, Yang Y-H, Ko Y-C (2006). Predictors of betel quid chewing behavior and cessation patterns in Taiwan aborigines. BMC Public Health.

[10] Ghani WM, Razak IA, Yang Y-H, Talib NA, Ikeda N, Axell T (2011). Factors affecting commencement and cessation of betel quid chewing behaviour in Malaysian adults. BMC Public Health.

[11] Lee C-H, Min-Shan Ko A, Warnakulasuriya S, Ling T-Y, Rajapakse PS, Zain RB (2012). Population burden of betel quid abuse and its relation to oral premalignant disorders in south, southeast, and East Asia: an Asian betel-quid consortium study. Am J Public Health.

[12] Little MA, Pokhrel P, Murphy KL, Kawamoto CT, Suguitan GS, Herzog TA (2014). The reasons for betel-quid chewing scale: assessment of factor structure, reliability, and validity. BMC oral health.

[13] Abrams DB, Niaura RS (1987). Social learning theory. Psychol Theor Drinking Alcohol.

[14] Ho Y-C, Yang S-F, Lee S-S, Chang Y-C (2017). Regulation of hypoxia-inducible factor-1α in human buccal mucosal fibroblasts stimulated with arecoline. J Formos Med Assoc.

[15] Khaja AH, Zwiad AA, Tarakji B, Gazal G, Albaba F, KalajI N (2016). The dependence on smokeless tobacco in the south Asian communities in East London. Glob J Health Sci.

[16] Kuo S, Lew-Ting C (2008). The health lifestyles of areca quid-chewing taxi drivers-an exploratory study from the viewpoint of social context. Taiwan J Public Health.

[17] Valliani A, Ahmed B, Nanji K, Valliani S, Zulfiqar B, Fakih M (2012). Use of smoke-less tobacco amongst the staff of tertiary care hospitals in the largest City of Pakistan. Asian Pac J Cancer Prev.

[18] Pakistan Buraeu of Statistics GoP. Distribution of districts. 2010 [cited 2017 02–08-17]. http://www.pbscensus.gov.pk.

[19] Bennett S, Woods T, Liyanage WM, Smith DL (1991). A simplified general method for cluster-sample surveys of health in developing countries.

[20] Piper ME, Piasecki TM, Federman EB, Bolt DM, Smith SS, Fiore MC (2004). A multiple motives approach to tobacco dependence: the Wisconsin inventory of smoking dependence motives (WISDM-68). J Consult Clin Psychol.

[21] Brandon TH, Baker TB (1991). The smoking consequences questionnaire: the subjective expected utility of smoking in college students. Psychological Assessment: A Journal of Consulting and Clinical Psychology.

[22] Berlin I, Singleton EG, Pedarriosse AM, Lancrenon S, Rames A, Aubin HJ (2003). The modified reasons for smoking scale: factorial structure, gender effects and relationship with nicotine dependence and smoking cessation in French smokers. Addiction.

[23] Herzog TA, Murphy KL, Little MA, Suguitan GS, Pokhrel P, Kawamoto CT (2014). The betel quid dependence scale: replication and extension in a Guamanian sample. Drug Alcohol Depend.

[24] Lee C-Y, Chang C-S, Shieh T-Y, Chang Y-Y (2012). Development and validation of a self-rating scale for betel quid chewers based on a male-prisoner population in Taiwan: the betel quid dependence scale. Drug Alcohol Depend.

[25] Zhu X, Zhu Q, Jiang C, Shen H, Wang F, Liao W, et al. Disrupted resting-state default mode network in betel quid-dependent individuals. Front Psychol. 2017;8 10.3389/fpsyg.2017.00084 PMC5276995 28194128

[26] Colby SM, Tiffany ST, Shiffman S, Niaura RS (2000). Are adolescent smokers dependent on nicotine? A review of the evidence. Drug Alcohol Depend.

[27] Hussain A, Zaheer S, Shafique K. Betel quid dependency and associated intrapersonal, interpersonal, and environmental factors among adolescents: a school-based cross-sectional survey. Subst Use Misuse. 2018:1–7. 10.1080/10826084.2018.1447964 29533120

[28] Bonilha AG, de Souza EST, Sicchieri MP, Achcar JA, Crippa JAS, Baddini-Martinez J (2013). A motivational profile for smoking among adolescents. J Addict Med.

[29] De Wilde KS, Tency I, Boudrez H, Temmerman M, Maes L, Clays E (2016). The modified reasons for smoking scale: factorial structure, validity and reliability in pregnant smokers. J Eval Clin Pract.

[30] Anjum MS, Srikanth MK, Reddy PP, Monica M, Rao KY, Sheetal A (2016). Reasons for smoking among the teenagers of age 14–17 years in Vikarabad town: a cross-sectional study. J Indian Assoc Public Health Dent.

[31] Perkins KA, Conklin CA, Levine MD (2008). Cognitive-behavioral therapy for smoking cessation: a practical guidebook to the most effective treatments: Taylor & Francis.

[32] Yap S-F, Ho P-S, Kuo H-C, Yang Y-H (2008). Comparing factors affecting commencement and cessation of betel quid chewing behavior in Taiwanese adults. BMC Public Health.

[33] Wang S-C, Tsai C-C, Huang S-T, Hong Y-J (2003). Betel nut chewing and related factors in adolescent students in Taiwan. Public Health.

